# Polysaccharides as Potential Anti-tumor Biomacromolecules —A Review

**DOI:** 10.3389/fnut.2022.838179

**Published:** 2022-02-28

**Authors:** Rui Guo, Min Chen, Yangyang Ding, Pengyao Yang, Mengjiao Wang, Haihui Zhang, Yuanqing He, Haile Ma

**Affiliations:** ^1^College of Food Science and Biological Engineering, Jiangsu University, Zhenjiang, China; ^2^The Laboratory Animal Research Center, Jiangsu University, Zhenjiang, China

**Keywords:** cancer, polysaccharides, anti-tumor activity, structure-activity relationship, molecular mechanisms

## Abstract

Cancer, as one of the most life-threatening diseases, has attracted the attention of researchers to develop drugs with minimal side effects. The bioactive macromolecules, such as the polysaccharides, are considered the potential candidates against cancer due to their anti-tumor activities and non-toxic characteristics. The present review provides an overview on polysaccharides' extraction, isolation, purification, mechanisms for their anti-tumor activities, structure-activity relationships, absorption and metabolism of polysaccharides, and the applications of polysaccharides in anti-tumor therapy. Numerous research showed extraction methods of polysaccharides had a significant influence on their activities. Additionally, the anti-tumor activities of the polysaccharides are closely related to their structure, while molecular modification and high bioavailability may enhance the anti-tumor activity. Moreover, most of the polysaccharides exerted an anti-tumor activity mainly through the cell cycle arrest, anti-angiogenesis, apoptosis, and immunomodulation mechanisms. Also, recommendations were made to utilize the polysaccharides against cancer.

## Introduction

Cancer, as one of the most life-threatening diseases, is mainly induced by irregularities in the cell division, which can be governed by genetic, environmental, and chemical factors (1). The global epidemiological survey (2018) estimated that the cumulative risks of morbidity and mortality due to cancer were 21.4 and 17.7%, respectively (2). Currently, surgery, radiation therapy, chemotherapy, targeted therapy, and immunotherapy have been developed to mitigate and treat cancer at different stages. However, these methods are still accompanied by drawbacks and side-effects, which could negatively impact the lives of patients with cancer (3). Therefore, developing new nontoxic drug molecules for cancer therapy is one of the major challenges of the researchers.

Bioactive compounds have been proven to be a potent anti-tumor agent, which exerts pleiotropic and synergistic effects with chemotherapy drugs, and targets multiple pathways of cancer (4). As one of the basic substances of life, polysaccharides are well-known for their anti-oxidation, immunomodulatory, anti-tumor, anti-inflammatory, and hypoglycemic activity (5–9). Since the discovery of anti-tumor activity in yeast polysaccharides in 1950, multiple types of polysaccharides with anti-tumor activity have been identified (10). It was demonstrated that *Trametes versicolor (Synn. Coriolus versicolor)* polysaccharides, *fucoidan*, and *sepia Ink* polysaccharides could be applied as adjuvant therapies of cancer, which usually possess low levels of toxicity and have high safety (11–13). Furthermore, polysaccharides could promote the recovery of patients with cancer (14, 15). It was observed that concomitant administration of *fungal* β*-glucan*, with chemo or radiotherapy, reduced the immune depression caused by such treatments or accelerated the production of white blood cells (16). Moreover, when co-administered with cyclophosphamide (CY), polysaccharides extracted from *Angelica sinensis* (AP) had cytoprotective effects on the hematopoietic and gastrointestinal tissues in patients with cancer (17). Therefore, it is of great significance to develop polysaccharides with better anti-tumor activities and minor side effects. Currently, natural polysaccharides have been extensively investigated, while the data concerning the mechanism of polysaccharides with anti-tumor activity and application of anti-tumor therapy were scattered. In this view, a review on natural polysaccharides, with anti-tumor activity and their mechanism for therapeutic application, is necessary. Moreover, the structure-activity relationship and the absorption and metabolism of polysaccharides with better anti-tumor activity have not been reviewed, which might limit their exploitation and utilization. Thus, the present review was conducted to summarize the extraction and the purification technology, the structure-activity relationships, and the absorption and metabolism of polysaccharides with better anti-tumor activity. Furthermore, the application of polysaccharides as anti-tumor therapy and their potential mechanisms were explored. Also, the recommendations were made to better utilize these natural products as an anti-tumor therapy. This review could contribute to a deeper understanding of polysaccharides as one type of new functional food or ingredients for human health.

## Extraction, Isolation, and Purification of Polysaccharides

With the application of polysaccharides in disease treatment and health improvement, the foremost step is to extract the natural product in its high purity and quality. Furthermore, natural polysaccharides experience physiochemical and functional changes during extraction, and thus, selection of an appropriate condition is necessary (18).

### Extraction

Currently, the most common methods for the extraction of polysaccharides include hot water extraction (HWE), ultrasound-assisted extraction (UAE), supercritical fluid extraction (SFE), ultrasound and microwave-assisted extraction (UMAE), and enzyme-assisted extraction (EAE). The effects of different extraction methods on polysaccharide activity are shown in Table 1. Therefore, the appropriate extraction methods could be screened out according to the characteristics of different polysaccharides. Furthermore, different extraction methods are helpful to prepare polysaccharides with better anti-tumor activity through changing the native polysaccharides' intrinsic viscosity and spatial conformation (19). Zhu et al. reported that the polysaccharides from *Cordyceps gunnii (C. gunnii) mycelia*, obtained by microwave-assisted extraction (MAE), could transform their spatial conformation to clumpy structures and exhibited better anti-tumor activity than by HWE and UAE (20). Meanwhile, the optimal extraction conditions for polysaccharides have played a key role in obtaining the extract of high quality with functional attributes. In an experiment of extracting *crude ginger* polysaccharides (GPs) with different extraction methods, an ultrasonic cell grinder extraction (UCGE) exhibited a stronger inhibitory effect (56.843 ± 2.405%) on three tumor cell lines, followed by HWE and EAE (21). It implies that polysaccharides with enhanced activities may be obtained by selecting suitable extraction methods.

**Table 1:** Advantages and disadvantages of extraction methods of polysaccharides.

**Methods**	**Advantages**	**Disadvantages**	**Resources**	**Condition**	**Improved functions**	**References**
HWE	Most widely used; easy-to-operate.	Long extraction time; poor effect of improving activity	*Dendrobium nobile Lindl*.	90°C, 120 min	Suppress the growth of the sarcoma 180 tumor cells	(22, 23)
			*Cymbopogon citratus*	99.66°C, 113.81 min	Increased the several cancer cells growth inhibition	(24)
SFE	High yield; hardly effects on the structure; less treatment time	High investment cost; low polarity of supercritical CO_2_;	*Dendrobium nobile Lindl*.	129.83°C, 16.71 min,1.12 Mpa	Anti-oxidant activities	(25, 26)
UMAE	High yield; low cost; less treatment time	Structural damage; strict reaction conditions;	*Camptotheca acuminata fruits*	Microwave power of 570 W, a fixed ultrasonic power of 50 W, 20 min	Inhibition on multiple cancerous cells	(27, 28)
			*Inonotus obliquus*	Microwave power of 90 W, ultrasonic power of 50 W, 40 kHz, 20 min	Anti-tumor activities	(29)
EAE	Reaction conditions are mild; Degrad polysaccharides for desirable fragments	Usually combined with other extraction methods	*Korean ginseng*	First treating with cellulase at 50°C for 24 h and then with α-amylase at 90°C for 24 h, pH 4.5	Immunostimulatory activity	(30, 31)
			*Lotus (Nelumbo nucifera Gaertn.) leaves*	Pectinase, 50°C, 48 h, pH 4.5–5.0	Immunostimulatory activity	(32)

### Isolation and Purification

The extract is a complex mixture of pigments, proteins, inorganic salts, lignin, bioactive compounds, etc. (33). The impurities need to be removed to identify the polysaccharides' structure and functional activity (34). Currently, the sevag method, trichloro trifluoroethane method, and trichloroacetic acid (TCA) methods could be used for the removal of protein, while anion exchange macroporous resin, hydrogen peroxide (H_2_O_2_), organic solvents successive rinse, and activated carbon adsorption could be used to remove pigments (35). There are several basic methods for purifying the polysaccharides mixture, as highlighted in Table 2.

**Table 2:** Advantages and disadvantages of isolation and purification methods of polysaccharides.

**Purification methods**	**Mechanism**	**Common types**	**Advantages**	**Disadvantages**	**References**
Solvent method	Respective solubility	Organic solvent	Simple operation; cost-effective	Co-precipitation; low reclamation; Solvent waste	(36, 37)
		Salting-out method			
Column chromatography	Charger property	Anion exchange column chromatography.	High separation efficiency; simple operation	Small loading amount; slow flow rate	(38, 39)
	Different molecular sizes and shapes	Gel Column Chromatography	High separation efficiency; widely used	Small loading amount; unsuitable for the separation of mucopolysaccharides	(40, 41)
	Molecule affinity property	Affinity chromatography	High separation efficiency; Easily for low-content polysaccharide	Difficult to find suitable ligands	(42, 43)
Membrane technology	Molecular weight distribution	Microfiltration	Simple operation; reaction conditions are mild	Low yield; long time-consuming; membrane fouling	(44, 45)

At present, the optimized combination of multiple methods may be more effective to obtain the highly-purified polysaccharides with multiple bioactivities (46). A binary system with hot water and supercritical CO_2_ has been developed to obtain extracts that are rich in anti-oxidant polysaccharides, which could increase the protection against oxidative damage induced by H_2_O_2_ tested in the HepG2 cells model (47). The purified water-soluble intracellular polysaccharides (IPSW-1) obtained by fractional ethanol precipitation, followed by ion-exchange and size exclusion chromatography, showed the prominent inhibitory effect on the HepG2 cell line with a 39% inhibition rate at the concentration of 70 μg/ml (48). The crude polysaccharides obtained from *the brown seaweed Sargassum pallidum* under supercritical CO_2_ extraction and ultrasonic-aid extraction were purified by membrane technology and diethylaminoethyl (DEAE) cellulose-52 chromatography. The polysaccharide fractions (SP-3-2) showed higher anti-tumor activity against the HepG2 cells than a blank control at 1 mg/ml concentration (49). After HWE (15 L/kg) for 2 h, followed by ethanol purification, polysaccharides from sporoderm-broken spore *of G. lucidum* (SGP) could improve the chemotherapy side effects by relieving a small intestinal barrier injury caused by paclitaxel (PTX) (50).

## The Mechanisms of Anti-Tumor Effects of Polysaccharides

**Figure 1:**
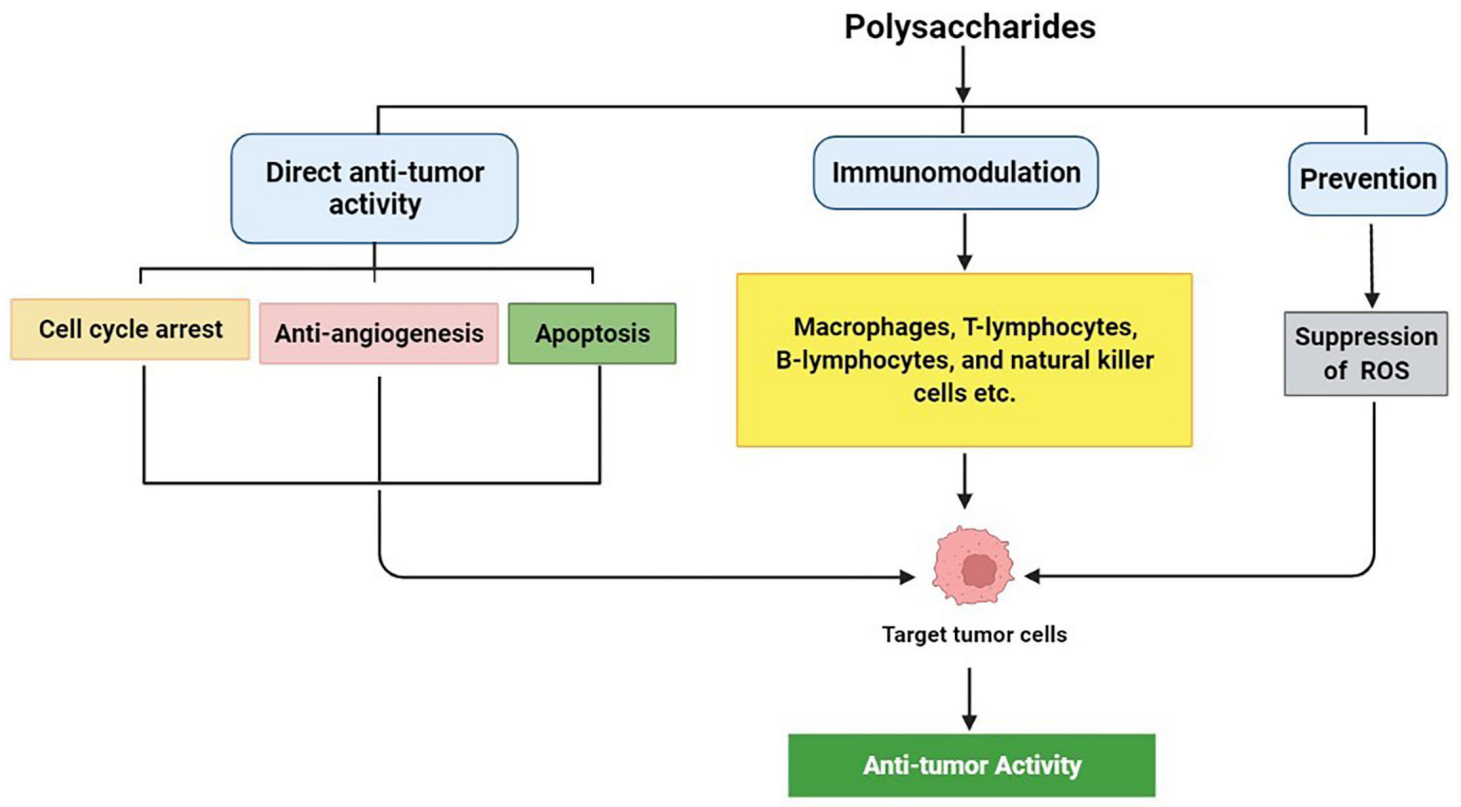
Summary of anti-tumor mechanisms of polysaccharides.

Numerous studies have shown that the most common mechanisms of anti-tumor polysaccharides include cell cycle arrest, anti-angiogenesis, and apoptosis, which exert direct tumor-killing capacities. Alternatively, immunomodulation could protect the immune system and induce indirect tumor-killing effects (51, 52). The mechanisms of polysaccharides' anti-tumor activity are shown in Figure 1.

### Cell Cycle Arrest

The cell cycle is defined as the whole process of cell division, including the interphases (G1, G0 S, and G2) and mitotic phases (M1 and M2). Numerous studies showed that the proliferation of tumor cells could be prevented by breaking the cell cycle at any point of the G1-S-G2-M phases (53). Cell cycle arrest at G0/G1 and G2/M phases could decrease the number of cells that subsequently enter the S-phase, which was attributed to inhibiting the cyclin-dependent kinases and activation of the cell cycle checkpoints, followed by the generation of subsequent deaths of the cancer cell lines (54). One recent study reported that the *Hawthorn (Crataegus.)* polysaccharides (HPS) treatment on human colon cancer cell line HCT116 for 12 h has decreased the mRNA expressions of Cyclin A1, Cyclin D1, Cyclin E1, and CDK (cyclin-dependent kinase) 1/2, resulting in cycle cell arrest in the S and G2/M phases (55). Jie et al. also discovered that *Auricularia polytricha* polysaccharides (APPs)-mediated cell cycle arrest at G0/G1 phase, mainly through inhibition of CDK/cyclin complexes formation, downregulating cyclin A, cyclin D, and CDK2, and activating p21 in a p53-dependent (p53 is an effective tumor suppressor) event in a human lung cancer cell line, which is the A549 (56). Thus, the regulation of cell cycle-related genes could inhibit the malignant growth of cancer cells by arresting the cell cycle progression.

### Anti-angiogenesis

Angiogenesis, the process of new blood vessel formation from pre-existing vasculature, is crucial for malignant tumor growth and tumor metastasis (57). It is widely recognized that inhibiting angiogenesis is an efficient strategy for treating various cancers in the early stages of tumor growth (58, 59).

Epidermal Growth Factor Receptor (EGFR) overexpression has been proven to be closely correlated with the pathogenesis of multiple malignancies (60). In addition, vascular endothelial growth factor (VEGF) played an important role in tumor-induced angiogenesis (61). Treatment with the water-soluble polysaccharide (PTP) (10, 20, and 40 mg/kg) prepared from the roots of *Polygala tenuifolia* could exert potential anti-angiogenesis effects by significantly downregulating the protein and mRNA levels of EGFR, VEGF, and CD34 (Hematopoietic progenitor cell antigen) in the tumor-bearing BALB/c mice, resulting in the suppression of tumor growth (62). Ren et al. conducted a tube formation assay and chick embryo chorioallantoic membrane (CAM) assay and demonstrated that *dandelion* polysaccharide (DP) could induce an anti-angiogenesis *in vitro* and *in vivo*. Furthermore, DP inhibited the expression of VEGF and HIF-1α (a hypoxia response protein) by targeting PI3K/AKT pathway, thereby downregulating the angiogenesis, which was believed to be a potential strategy to control tumor growth (63).

### Apoptosis

Apoptosis is a physiological process of programmed cell death that plays an important role in cancer treatment (52). Multiple polysaccharides have been recognized to induce apoptosis of cancer cells. A group of cysteine proteases (caspase-3, caspase-8, and caspase-9), which are key executioners for propagating the apoptotic signal, could trigger caspase-dependent apoptosis (64). In the case of *Cordyceps sinensis* polysaccharide (CSP), the activation of caspase-8 and caspase-3 induced apoptosis in human colon cancer cell line HCT116 (65). Also, treatment on A549 cells with Hedyotis diffusa polysaccharide (HDP) for 48 h could promote the release of cytochrome C from mitochondria into the cytosol, increase the pro-apoptotic Bax protein expression, unchanged anti-apoptotic Bcl-2 protein, and increase the Bax/Bcl-2 ratio in A549 cells. Moreover, the activated caspase-9 and −3 contribute to the mitochondrial-dependent apoptotic pathway (66). Previous studies also demonstrated that the alcohol-soluble polysaccharides of the rhizome of *Atractylodes macrocephala Koidz* (APA) promoted apoptosis on human esophageal cancer cells, the Eca-109, by a mitochondrial pathway (67). Furthermore, Fas/Fas ligand (Fas/FasL) played a significant role in tumorigenesis (68). The PGL (*Gracilariopsis lemaneiformis polysaccharide*) was found to exert a potential effect against a neoplastic disease by upregulating the Fas/FasL's protein and mRNA expressions, resulting in a Fas/FasL-dependent apoptosis in tumor cells (69).

### Immunomodulation

It has been proven that the anti-tumor activity was closely related to immunomodulation (70). The polysaccharides could activate macrophages, T-lymphocytes, B-lymphocytes, natural killer cells, and cytokines that are closely related to the killing of the tumor, such as tumor necrosis factor (TNF-α), interferon (IFN-γ), and interleukins (IL-2, IL-4, IL-6, and IL-12) (71). The anti-tumor immune response regulated by macrophages may be the first line of defense against tumors (59). Multiple polysaccharides could induce robust immune responses against tumors. They may work independently to give anti-tumor activity through the upregulation of the surface accessory molecules of resident macrophages and dendritic cells (DCs) to promote secreting cytokines, which increased the serum IL-2 levels. This is observed in the case of *Epimedium koreanum Nakai* polysaccharides (EPS) treatment (72). Moreover, *Artemisia argyi* polysaccharides (FAAP-02) were reported to improve anti-tumor activity by promoting the production of lymphocyte, TNF-α, IL-2, IL-4, IL-6, and IL-12. Thus, FAAP-02 could improve the 5-fluorouracil's side-effects when co-administered with 5-fluorouracil (73).

### Other Pathways

In addition to the mechanisms discussed above, the nitric oxide (NO) pathway may also be a mechanism of the anti-tumor polysaccharides. Several polysaccharides are reported to stimulate macrophages to produce NO, which could directly act on tumor cells *via* upregulation of inducible NO synthase (iNOS) activity (51). Reactive oxygen species (ROS) involved in the transformation, tumor survival, proliferation, invasion, and metastasis of cancer cells and polysaccharides could suppress ROS to prevent activation of pro-tumorigenic signaling pathways (74, 75). The ROS-centered pathways include mitochondrial autophagy, mitogen-activated protein kinase (MAPK), and transcription factor-related pathways like nuclear factor kappa-B (NF-κB) and hypoxia-inducible factor (HIF). These pathways could be utilized with or without the further involvement of inflammatory and death receptor pathways (76). This may be helpful in the prevention of cancer in healthy individuals, as observed with the administration of *Ganoderma lucidum* polysaccharide (77). So far, the molecular mechanisms of polysaccharides are mostly investigated *in vitro* or animal models, and further research on clinical investigations is needed (53).

## Structure-Activity Relationship and Bioavailability of Polysaccharides

Structural diversity (including primary, secondary, tertiary, and quaternary structures) is one of the important factors affecting the anti-tumor activity of the polysaccharides (78). The primary structure includes the composition of monosaccharides, the types of glycoside bonds, the order of connection, heteropolar carbon configuration, molecular weight, position and length of branches, and degree of substitution (79). The secondary structure of polysaccharide chains (mainly including helical structure) is described as the conformation of individual monosaccharide residues and the geometry of their linkage to each other by the glycosidic bonds (80). The tertiary and quaternary structures are the arrangement of single units (based on secondary structure) within a complex built by non-covalent interactions (hydrogen bonds, van der Waals forces, *etc*.) (78). The main findings of the different studies concerning the structure, modification, and bioavailability of the anti-tumor polysaccharides will be summarized in this section. The types and structural diversity of polysaccharides are shown in Table 3. However, the characteristics of the complex structure of the polysaccharides require a lot of work by combining the various analytical methods and the more precise instruments. There are few studies on the analysis of the higher space structure of polysaccharides, and further research on the structure-activity relationships needs to be conducted (81).

**Table 3:** Types and structural diversity of polysaccharides.

**Structure types**	**Composition and sequence of monosaccharide**		**Configuration of each monosaccharide residue**	**Anomeric form of each glycosidic bond**	**Types of linkages**	**Spatial configuration**	**Modifying groups**
	Homopolysaccharide	Arabinose; xylose; mannose; glucose; galactose;	D-	α-	α-(1 → 2); α-(1 → 3); α-(1 → 4); α-(1 → 5); α-(1 → 6);	Single-helix; triple helix; random coil	Sulfate group; acetyl group; phosphate group; methyl group;
	Heteropolysaccharide	Rhamnose; xylose; glucose; mannose; arabinose; galactose	L-	β-	β-(1 → 3); β-(1 → 4); β-(1 → 6)		

### Structure-Activity Relationship of Polysaccharides

The structural variability of a polysaccharide, such as the location of the monosaccharide residues, the position of glycosidic linkages, and the sequence of monosaccharide residues, are closely associated with the biological activity (59). Currently, the structure of the polysaccharides could be preliminarily analyzed *via* various methods, including molecular weight determination, spectral analysis, monosaccharide composition analysis, *etc*. A detailed investigation of the chemical structure and chain conformation could be the hotspot of structure-activity research on anti-tumor polysaccharides. The polysaccharides, with the main chain consisting of β-(1 → 3)-glucans and additional β-(1 → 6) branches, could enhance their immunostimulatory and exhibit a prominent anti-tumor activity by promoting the interaction with specific receptors (82). The *A. pullulans*-derived β-glucan could exhibit an anti-tumor activity by enhancing the interferon-gamma (IFN-γ) production and the NK cell activity by triggering the intestinal immunity (83). In addition, the *Grifola frondosa* polysaccharide, consisting of β-(1 → 3)-linked glucan branched with β-(1 → 6) glucosides, could be used as an immune-adjuvant therapy for cancers (84). The presence of hydrophilic (polyhydroxylated) groups, located outside on the surface of the triple helix, could also be important for the anti-tumor activity by increasing the immune-competent cell activity (70). Zhang et al. proved that the triple-helix conformation plays an important role in enhancing the anti-tumor effects of the lentinan. The triple-helix sample effectively inhibited the growth of Sarcoma 180, a solid tumor with the inhibition ratio of 49.5%, close to that of 5-fluorouracil (50.5%). In contrast, the bioactivity (12.3%) of its single flexible chains almost disappeared (85). The presence of branches could regulate different chain conformations, and the degree of the branch (DB) was also found crucial for polysaccharides' activity (84). It was discovered that the anti-tumor activity has rapidly dropped when the branching reached up to 9.3%, then, slightly increased as the branching degree of M-levan further decreased in HepG2 cell lines (86).

### Modification and Anti-tumor Activity

The inherent structure of polysaccharides may partly limit its anti-tumor activity. The structural changes (including molecular weight, types of substituent groups, dimensional structure, *etc*.) caused by the molecular modification could improve the bioactivities of polysaccharides and promote the application of polysaccharides (15, 87). Molecular modification could hold changes in the steric hindrance and electrostatic repulsion, while the flexion and the extension of polysaccharide chains and the water solubility impact the bioactivities (88). In this section, the current modification methods of polysaccharides were compared (Table 4), which exerted different effects on the activity of polysaccharides.

**Table 4:** The different modifications of polysaccharides.

**Modifications**	**Methods**	**Mechanism**	**Functional changes**	**References**
Physical modification	Ultrasound	Cavitation effect induced the solute component structure changes.	Molecular degradation; the degree of polymerization and molecular weight were decreased; anti-virus, an-titumor and anti-oxidation activities were improved.	(89)
Chemical modification	Sulfuration	Replace hydroxyl group by sulfuric acid groups on the residues of polysaccharides.	Molecular weight was changed; solubility, anti-virus, anti-tumor and anti-oxidation activities were improved.	(90)
	Carboxymethylation	Replace hydroxyl group by carboxymethyl group on the residues of polysaccharides.	Water-solubility, anti-virus, and anti-tumor activities were improved.	(91)
	Acetylation	Replace hydroxyl group by acetyl group on the residues of polysaccharides.	Water-solubility and immunostimulation effects were improved.	(92)
	Phosphorylation	Replace hydroxyl group by phosphate groups on the residues of polysaccharides	Water-solubility, anti-virus and anti-oxidation activities were improved.	(93)
Biological modification	Enzymic method	Enzyme-catalyzed	Molecular degradation; the degree of polymerization and molecular weight were decreased; anti-oxidation activities was improved.	(94)

Currently, the modification methods mainly focus on the chemical modification of polysaccharides. It was shown that sulfate modification of polysaccharides could enhance the anti-tumor activity by increasing their immune-stimulating activity (98). Jiang et al. reported that the polysaccharide from *Dimocarpus longan Lour*. (LP1) and its sulfated derivative (LP1-S) stimulated the proliferation of lymphocytes and the macrophage function, which play an important role in the anti-tumor activity, while the LP1-S had the better immunopotentiation (99). Furthermore, phosphorylation is the most common modification of increasing the anti-tumor activity (100, 101). After phosphorylation of polysaccharides, the charged phosphate groups could improve water solubility, change molecular weight, and modify chain conformation of polysaccharides, resulting in the alteration of biological activities (102). The phosphorylated polysaccharides (P-DIP), obtained from the natural polysaccharides in *Dictyophora indusiata* (DIP), also showed significant inhibitory effects on the growth of MCF-7 and B16 tumor cells, while DIP had no inhibiting effects on them (103). The selenylation polysaccharides can increase antioxidant effects and reduce cancer risk (104). The selenized polysaccharides from *alfalfa* (Se-RAPS-2) has superior anti-tumor activity than the two native polysaccharides (RAPS-1 and RAPS-2), and the inhibition ratios of RAPS-1, RAPS-2, and Se-RAPS-2 on HepG2 cells were 30.35, 19.81, and 38.70% at a concentration of 100 μM, respectively (105).

### Absorption and Metabolism of Polysaccharides

The active ingredient, either in food or medicine, commonly works after being absorbed and metabolized. The oral route is one of the most common routes of administration for food and medicine. Polysaccharides could be digested and absorbed in the gastrointestinal tract after oral administration, and the upper small intestine is the main absorption site (106). Zhang et al. found that Cyanine7 amine-labeled polysaccharides, extracted from *Smilax china L*. (Cy7-SCLP), could be diffused through the mucus barriers and absorbed in the small intestine. Furthermore, Cy7-SCLP could be absorbed after oral administration through endocytosis process mediated by macropinocytosis pathway and clathrin- and caveolae (or lipid raft)-related routes, and then circulated into blood (107). A modified citrus pectin (MCP) has the properties of a low-molecular-weight degree of esterification, which could be absorbed from the small intestinal epithelium, and then enter the circulation. Also, the MCP is Galectin-3 inhibitors, thus, conducive to decelerating the cancer metastasis (108). Some polysaccharides for the energy supply are directly absorbed into the small intestine and metabolized. Cao et al. discovered that *Poria cocoa* polysaccharide was mainly degraded into stable oligosaccharide fragments in gastric juice, and then, absorbed in the intestine in rats (109).

Fermentation in the large intestine is the main metabolism method of complex polysaccharides (including non-starch polysaccharides, resistant starches, and dietary fiber in food), which could not be digested directly by humans (110). The gut microbiome also played an important role in digesting polysaccharides (111). The gut bacteria could degrade the complex polysaccharides to generate gas, heat, and short-chain fatty acids like butyrate and propionate (112, 113). This metabolic process could improve immune functions and inhibit the occurrence of tumors. In addition, some degradation products of polysaccharides had protective metabolites, which could reduce the risk of cancer. For instance, the butyrate could modulate the action of PKC (protein kinase C) by activating the AP-1 signaling pathway, which could promote the apoptosis of colon cancer cells (114).

However, the multiple polysaccharides are large and complex molecules that cannot be easily degraded during human digestion. Their large size prevents intestinal absorption, resulting in their poor bioavailability and anti-tumor activity (111, 115). The factors including bioaccessibility, absorptivity, and metabolic conversion rate usually limit the oral bioavailability of active ingredients (116). Therefore, various research was conducted on modifications of polysaccharides to improve their bioavailability, as summarized in Table 5 (117–119).

**Table 5:** Methods for improving the bioavailability of polysaccharides.

**Method**	**Common types**	**Function**	**References**
Carriers	Nanoparticles (NPs)	NPs are endowed with a relatively large (functional) surface which is able to bind, adsorb and carry other compounds.	(95)
Absorption promoters	Chitosan (CS)	CS has the mucoadhesive feature which increase the paracellular permeability.	(96)
	Trimethyl chitosan chloride (TMC)	TMC forms complexes with anionic macromolecules and gels or solutions with cationic or neutral compounds	(97)

## Application of Polysaccharides with Anti-Tumor Activities in Drugs and Food

The polysaccharides' application in food and drugs is a research hotspot due to their significant biocompatibilities and anti-tumor activity. This section focuses on the applications of anti-tumor polysaccharides that were studied in the last 5 years as drugs and food.

### Drugs

Chemotherapy is one of the main therapies for cancer treatment. Current chemotherapeutic drugs are accompanied by larger side effects, such as impairing the immune system, drug resistance, and toxicity to normal tissues (120). In contrast, polysaccharides could synergistically fight tumors with chemotherapy drugs and improve the injury caused by the surgery and radiotherapy (121, 122).

Lentinan could work in tandem with other chemical drugs, and S-1/paclitaxel/lentinan; the S-1/CDDP/lentinan, S-1/lentinan, and superfine dispersed lentinan (SDL) chemotherapies for the treatment of advanced gastric cancer, pancreatic cancer, colorectal cancer, and hepatocellular carcinoma have been developed to decrease the incidence of adverse effects of chemotherapies, to improve immunity, and to extend patients' lives (123). Furthermore, Ru (flavonoid rutin), which is non-covalently complexed with fucoidan (Fu), was proved to induce apoptosis in HeLa cells *via* promoting nuclear fragmentation, ROS generation, and mitochondrial potential loss without significant impacts on normal cells. Hence, the Ru-Fu complex can be used as a new anti-carcinogen for cervical cancer and could be further tested on other cancer cells (124).

The polysaccharides, with the abilities of intrinsic immunomodulation, biocompatibility, biodegradability, low toxicity, and high safety, have been used as a type of adjuvants for vaccines against cancer, which could facilitate the immunogenicity of antigens, induce stronger immune responses in populations that are responding poorly to vaccination, and decrease the dosage and production cost of vaccine (125). The *Ganoderma lucidum* polysaccharides, which served as a potential natural therapeutic agent, were used to suppress protein synthesis and growth of tumor cells *via* disturbing the survival and proliferative signaling pathways (126). In 2010, the State Food and Drug Administration (SFDA) in China approved *Ganoderma sinense* polysaccharide (GSP) tablet as an adjunctive therapeutic drug for treating leukopenia and hematopoietic injury count caused by radiation therapy and chemotherapy (127).

Currently, polysaccharides (or derived polysaccharides) with nanoparticles (nanogels) could be synthesized *via* polyelectrolyte complexation (PEC), self-assembly, covalent cross-linking, and ionic cross-linking methods. The polysaccharide-based nanogels were widely used in drug delivery systems (128). To improve drug absorption and stabilize drug components, chitosan could be used as polymer-drug conjugates or chitosan-based nanocarriers, which further increased drug targeting and executed better anti-tumor effects with lesser side effects, and supported the sustained release of anti-tumor drugs in the cancer tissues (129, 130). Fucoidan has been involved in drug delivery systems (DDS). Fucoidan, in conjugation with chemotherapeutic drugs, e.g., doxorubicin (DOX), could increase the toxicity to cancer cells (around 25 %) without showing any toxicity to normal cells (131).

### Functional Foods

Also, polysaccharides can be used as ingredients of functional food. *Inulin* (INU) polysaccharides have physiochemical properties of hydrophilicity, low molecular weight, and resistance against degradation in the stomach and intestinal fluids. The INU has been approved by the Food and Drug Administration in India for improving the nutritional values of beverages, yogurts, biscuits, and spreads as dietary fibers (132). The *Stigma maydis* polysaccharides have been used to produce multiple functional food, including stigma maydis beverage, stigma maydis tea, stigma maydis oral solution, and stigma maydis tablet, which have the advantages of inhibiting blood glucose levels, improving immunity, and enhancing the anti-tumor activities (133).

Present challenges hinder the wider application of polysaccharides. Many studies on safer usage, requisite toxicity, and potential mutagenicity must be conducted. Hence, the medical applications of some polysaccharides remained in the stages of clinical trials. Despite possessing potential abilities, it is necessary to pay attention to the interactions between polysaccharides and other components, thus, hindering their bioactivity. For example, it was discovered that the anti-tumor activity might be reduced when the lentinan and other biopolymers interacted with carrageenans (134). After overcoming the issues of preparation, quality standards, route of administration, and other technical problems, polysaccharides could open new applications in cancer treatment (122, 125, 135).

## Conclusion

Polysaccharides could play an important role in cancer therapy, and new classes of polysaccharides with anti-tumor activity were constantly being explored in recent years. This review clearly shows that polysaccharides could be used as promising adjuvants or functional foods for cancer therapeutics through multiple pathways.

However, some challenges hinder the applications of polysaccharides. A comprehensive study on the structure-activity relationship and absorption mechanism of polysaccharides is helpful in the development of polysaccharides as commercial products. Some action mechanisms of anti-tumor polysaccharides have been illustrated in *in vitro* and animal experiments; however, clinical studies need to be conducted to better understand its effects and mechanisms. Nevertheless, more experiments and clinical studies on the practical application of polysaccharides are necessary to be conducted. It could be expected that polysaccharides are of great significance as tumor therapy and for improving human health.

## Author Contributions

RG: writing — original draft. MC and YD: writing — review and editing. PY: visualization. MW: investigation. HZ: supervision. YH: review and project administration. HM: funding acquisition and project administration. All authors contributed to the article and approved the submitted version.

## Funding

This work was supported by grants from Jiangsu University Foundation of China (No. 07JDG016), the Laboratory Animal Association Foundation of Jiangsu (No. DWXH201910), and the Key Technology R&D Program of Zhenjiang, China (No.SH2019011).

## Conflict of Interest

The authors declare that the research was conducted in the absence of any commercial or financial relationships that could be construed as a potential conflict of interest.

## Publisher's Note

All claims expressed in this article are solely those of the authors and do not necessarily represent those of their affiliated organizations, or those of the publisher, the editors and the reviewers. Any product that may be evaluated in this article, or claim that may be made by its manufacturer, is not guaranteed or endorsed by the publisher.
